# Agronomical Parameters, Sugar Profile and Antioxidant Compounds of “Catherine” Peach Cultivar Influenced by Different Plum Rootstocks

**DOI:** 10.3390/ijms15022237

**Published:** 2014-02-03

**Authors:** Carolina Font i Forcada, Yolanda Gogorcena, María Ángeles Moreno

**Affiliations:** Departamento de Pomología, Estación Experimental de Aula Dei (CSIC), Apartado 13034, Zaragoza 50080, Spain; E-Mails: carolffont@gmail.com (C.F.F.); aoiz@eead.csic.es (Y.G.)

**Keywords:** *Prunus persica*, fruit quality, sugars, High Performance Liquid Chromatography (HPLC), antioxidant compounds, vitamin C

## Abstract

The influence of seven plum rootstocks (Adesoto, Monpol, Montizo, Puebla de Soto 67 AD, PM 105 AD, St. Julien GF 655/2 and Constantí 1) on individual and total sugars, as well as on antioxidant content in fruit flesh of “Catherine” peaches, was evaluated for three years. Agronomical and basic fruit quality parameters were also determined. At twelve years after budding, significant differences were found between rootstocks for the different agronomic and fruit quality traits evaluated. The Pollizo plum rootstocks Adesoto and PM 105 AD seem to induce higher sweetness to peach fruits, based on soluble solids content, individual (sucrose, fructose and sorbitol) and total sugars. A clear tendency was also observed with the rootstock Adesoto, inducing the highest content of phenolics, flavonoids, vitamin C and relative antioxidant capacity (RAC). Thus, the results of this study demonstrate the significant effect of rootstock on the sugar profile and phytochemical characteristics of peach fruits. In addition, this work shows the importance of the sugar profile, because specific sugars play an important role in peach flavour quality, as well as the studied phytochemical compounds when looking for high quality peaches with enhanced health properties.

## 1. Introduction

Peach [*Prunus persica* (L.) Batsch] is one of the most important deciduous fruit trees in the world after apples and pears. This crop is economically important with a production of around 20.2 million tons in 2010 and with a cultivated area approximately of 1.6 million ha [1]. China, the Mediterranean area (Italy and Spain) and United States are the four top producers of peach in the world. In Spain, the peach production (around 1.4 million tons in 2010) contributes to the major production among *Prunus* species (including apricots, European and Japanese plums, peaches and sweet cherries) and pome fruits (apples and pears). Peach production represents 4.5% of total Spanish horticultural products [2].

Commercial peach trees are usually composed of two genetically different parts: a scion cultivar (aerial part) and a rootstock (root system). The use of rootstocks is mainly directed to overcome soil and disease problems to which peach scions have limited or no resistance, as root asphyxia and iron-chlorosis deficiency in compact and calcareous soils, respectively [3]. Plum rootstocks are more tolerant to compact soils and waterlogging than other species of *Prunus* L., a fundamental reason for their use as rootstocks for peaches. In addition, some of them provide greater tolerance to iron-chlorosis deficiency and to soil-borne pathogens, such as fungi and root-knot nematodes, so common in many peach-growing regions of the Mediterranean area.

The selection of an appropriate rootstock is important because some factors such as yield, vigour or fruit quality depend on the selection of the right scion-rootstock combination. The rootstock effect on tree growth, survival, yield or fruit weight is well known for the most commonly used rootstocks in the peach industry [3–5]. However, most vigorous and high-yielding peach-based rootstocks have been shown to induce lower fruit quality to the budded cultivars, probably due to the higher strength of vegetative growth *versus* fruit quality [5]. Thus, different genetic background of rootstocks can affect fruit quality, indicating that vigour and yield are not the only parameters affected by rootstocks [6,7].

In the fruit industry, there is an increasing interest in the nutritional value of fruit quality traits [8,9] because they represent multiple factors relevant to human health. Sugar profile, vitamin C, antioxidant capacity and phenolic compounds are the most important phytochemical compounds that could play an important role in gastrointestinal health [10], and help to minimize the effect of oxidative stress and diseases such as cancer or cardio-vascular disease [11]. The most abundant sugar in ripe peaches and nectarines is sucrose, followed by glucose, fructose, and finally sorbitol. Sucrose is important as a sweetener and energy source [12]. Fructose is sweeter than sucrose and glucose, and sorbitol is more beneficial than others with regard to diet control [10].

Fruit quality depends mainly on the scion genotype [13], but also may be influenced by the rootstock [14,15] and climatic conditions. The effect of different cultivars on sugar profile, phenolics content and antioxidant capacity of different type of peaches has been reported [16–19]. The effect of different peach-almond hybrid rootstocks on individual sugars of a peach and a nectarine cultivar has been reported [20]. Also, Remorini *et al.* [4] showed the effect of different rootstocks on some phytochemical compounds, including the total antioxidant capacity of Flavorcrest peaches. The significant effect of cultivars and rootstocks on these compounds and the increasing consumer interest in functional foods is demonstrated through the priority to evaluate the effect of new rootstocks on antioxidant compounds and other nutritional properties as presented here.

A key to the commercial expansion of peach production is the promotion and maintenance of the highest possible standards of fruit quality. Since rootstock strongly affects agronomic parameters of the budded cultivars, it is also advisable to know the role of rootstocks on the fruit quality and the relationship between agronomical and quality traits. Until now, fruit quality effects have been much more studied in cultivars [9] than in rootstocks and little is known about the influence of rootstocks with different genetic origin on sugar profile and phytochemical composition of the fruit.

At the Experimental Station of Aula Dei (Consejo Superior de Investigaciones Científicas, CSIC, Zaragoza, Spain), a breeding program of *Prunus* rootstocks adapted to Mediterranean conditions is under process. Within this program [21], local Spanish plums named Pollizo (*Prunus insititia*) were included for clonal selection as multi-purpose rootstocks for different *Prunus* species [22], but especially for peach trees grown in heavy and calcareous soil conditions. In addition, it is commonly assumed that Pollizo plums induce higher fruit quality to peaches than the most frequently used peach × almond hybrids or peach seedlings. Thus, the present study aims to evaluate the effect of seven plum rootstocks of different genetic origins, among them five Pollizo plums, on agronomical and fruit quality traits of peaches.

## 2. Results and Discussion

### 2.1. Fruit Quality Traits Evaluation

Table 1 shows factors affecting agronomical and fruit quality parameters in the “Catherine” peach cultivar. Rootstock influenced the levels of trunk-cross sectional area (TCSA), yield, cumulative yield, yield efficiency, fruit weight (FW), soluble solids content (SSC), flesh firmness (FF), a ripening index (RI), sucrose, fructose, sorbitol, total sugars, phenolics, flavonoids, vitamin C and relative antioxidant capacity (RAC). Similarly, the significant effect of year was found for TCSA, yield, cumulative yield, yield efficiency, FW, SSC, FF, titratable acidity (TA), RI, sugars (glucose, fructose and sorbitol), phenolics and anthocyanins. The year-to-year variation in fruit quality parameters may be explained by the differences in annual temperatures and crop load over the three years of the study. ANOVA results showed the absence of interaction between rootstock and year for all traits evaluated. This could indicate that rootstocks had consistent effects on “Catherina” peaches and their fruit quality traits.

Regarding basic fruit quality parameters (Table 2), the average of the three years of study shows that Constantí 1 induced the highest value to “Catherina” fruits for FW (g), although no significant differences were found when “Catherina” was budded on the Pollizo Adesoto. In contrast, P. Soto 67 AD, PM 105 AD and GF 655/2 rootstocks showed lower values of FW, but differences were not statistically significant from Monpol and Montizo. Regarding SSC (°Brix), fruits of “Catherina” on PM 105 AD showed the highest value, although no significant differences were found with Adesoto and Monpol. The lowest values were induced by Montizo and GF 655/2, but they did not significantly differ from Constantí 1. Orazem *et al.* [7] evaluated the performance of “Redhaven” peach cultivar grafted on eleven *Prunus* rootstocks. They observed that Adesoto rootstock induced higher values on FW and SSC to “Redhaven” fruits when compared to five other different plums and five peach-based rootstocks. Regarding firmness (N), no significant differences were found among rootstocks in the average value for the three years. In the absence of differences for firmness and TA, higher RI is due to the ability of specific rootstocks to induce higher SSC [5,17].

The sucrose, glucose, fructose and sorbitol contents of “Catherine” peaches (Table 3) were analyzed separately by High Performance Liquid Chromatography (HPLC) because they play an important role in peach flavour quality [23,24]. Sucrose was the sugar present at the highest concentration, as previously reported in peaches and nectarines [17,19] followed by fructose, glucose and sorbitol. Their levels differed significantly among rootstocks (Table 3), as showed by Albás *et al.* [20] comparing three peach-almond hybrids, and Orazem *et al.* [7] and Orazem *et al.* [25] studying different peach-based and plum rootstocks.

For sucrose (Table 3), the Pollizo Adesoto induced the highest average content on “Catherina” fruits, although no significant differences were found with PM 105 AD. Differences were not significant among the later and the rest of the other rootstocks. Values of sucrose ranged among rootstocks and years from 61.0 to 72.1 g kg^−1^ FW. For glucose, no significant differences were found among rootstocks. Fructose content was higher on Adesoto and lower on PM 105 AD but not significantly different from the other rootstocks. The fructose has been shown to be sweeter than sucrose by as much as between 1.75–1.80 times [26], and both of them have been shown to have beneficial effects on gastrointestinal health [10]. Consequently, Adesoto could have an additional value to be considered in the future. For sorbitol content, Adesoto again showed the highest content and Montizo, GF 655/2 and Constantí 1 the lowest, but they did not differ significantly from the other rootstocks. The sorbitol content varied greatly among rootstocks ranging from 3.0 to 6.2 g kg^−1^ FW. Colaric *et al.*, reported sorbitol content as the attribute most related to peach aroma and taste [27]. In addition, it is more beneficial than other sugars with regard to diet control, dental health and to avoid gastrointestinal problems, and it can be used as a glucose substitute [28]. Similar results were found on total sugars content, calculated as the sum of sucrose, glucose, fructose and sorbitol contents. Adesoto induced the highest value while Montizo, P. Soto 67 AD, GF 655/2 and Constantí 1 showed the lowest. The other two rootstocks (Monpol and PM 105 AD) did not differ significantly from all of them. Total sugar content ranged from 80.8 to 96.2 g kg^−1^ FW. Results for Adesoto agree with the work of Orazem *et al.* [7] and Orazem *et al.* [25] showing that Adesoto rootstock induced higher values on individual and total sugars compared with the other rootstocks. This is an interesting result as these sugars strongly affect peach flavour quality [23].

Other phytochemical traits (Table 4) seem to follow the same tendency of individual and total sugars content. In the average of the three years of study, the highest value for total phenolics was induced by Adesoto rootstock, although no significant differences were found with PM 105 AD and P. Soto 67 AD. The lowest value was found on Constantí 1, but it did not differ significantly from Monpol. The phenolics content varied greatly among rootstocks and years ranging from 22.8 to 34.3 mg GAE/100 g FW. Values are within the range reported for peach flesh in the literature [13,18,19]. Regarding flavonoids content, Adesoto also induced the highest value, but no significant differences were found with PM 105 AD and Montizo. In contrast, GF 655/2 and Constantí 1 induced the lowest values, although they did not significantly differ from P. Soto 67 AD and Monpol. Values for flavonoids ranged from 6.6 to 11.7 mg CE/100 g FW. Other authors have reported values of flavonoids in this range [13,18]. Higher values of flavonoids content in peaches and nectarines are found in the literature when skin is included in the sample [25] due to unequal distribution of phenolic compounds in the flesh. However, since peach skin is not usually eaten by consumers, it takes no part in the human diet. Concerning anthocyanins content, no significant differences among rootstocks were found. In “Catherina” peaches, anthocyanins are present in lower concentrations than in fruits of cultivars with more intensive red coloration [19]. For vitamin C, Adesoto and PM 105 AD induced the highest values, although they did not differ significantly from Montizo, where the lowest values were induced by GF 655/2 and Constantí 1. Values ranged from 5.4 to 9.6 mg ASA/100 g FW. They were in the same range as previously reported for vitamin C contents in peach flesh, namely, 1–14 mg of AsA/100 g of FW [18,19]. Finally, in a similar way to phenolics and flavonoids, the highest content of RAC was also induced by Adesoto, although no significant differences were found with PM 105 AD and Monpol. The lowest value was found on Constantí 1, but it did not differ from Montizo, P. Soto 67 AD and GF 655/2. The RAC content varied among rootstocks and years ranging from 345.6 to 502.2 μg Trolox/g FW. The antioxidant capacity observed in this study was in the range previously reported for peach flesh (100–1000 μg of Trolox/g of FW) [18,19]. Antioxidant compounds have been influenced by rootstocks, as previously reported [25]. The interaction between rootstocks and cultivars influences the levels of phytochemical traits, and this could have a crucial impact on the health promoting properties of peach fruit [13]. Thus, some cultivars that contain high levels of beneficial traits could be increased depending of the rootstock.

In summary, in this study, two Pollizo selections (Adesoto and PM 105 AD) induced the highest fruit quality, regarding SSC, sugars contents and phytochemical compounds, when compared to other Pollizo plums, another *P. insititia* (GF 655/2) or *P. domestica* (Constantí 1) plums as rootstocks for “Catherine” peach cultivar. In addition, Adesoto induced an intermediate level of tree vigour and greater yield efficiency (data not shown), being one of the most high-yielding rootstocks in this trial, in good agreement with Orazem *et al.* [7], who also reported that Adesoto resulted in the best fruit quality (SSC, individual and total sugars levels). The present work confirms the good performance of this rootstock, and jointly with PM 105 AD emphasize the interest of some Pollizo rootstocks to reach higher fruit quality peaches.

### 2.2. Correlations between Agronomical Parameters and Fruit Sugars Content and Phytochemical Traits

Some statistically significant correlations were found among agronomical parameters, sugars profile and phytochemical traits related to fruit quality (Table 5). Yield was positively correlated with TCSA, yield efficiency, fruit weight and anthocyanins, but negatively correlated with SSC, sucrose, flavonoids, vitamin C and RAC. Negative correlations between yield and some fruit components, such as SSC or sucrose, can be due to the sink competition of more fruits in development compared to fruit quality [29]. Fruit weight was significantly and positively correlated with SSC, sucrose, glucose, total sugars, phenolics and flavonoids as also reported by Cantín *et al.* [30] in different peach and nectarine progenies. That correlation is probably due to the fact that the rate of fruit growth is determined by the amount of available carbohydrates [29].

All individual sugars were positively and highly correlated with total sugars content. Correlation values between total sugars and glucose or fructose were also higher than between total sugars and sorbitol. Also, significant correlation values among sucrose, glucose and fructose were higher than values between these sugars and sorbitol. Previous studies on fruit sugar content in peaches and nectarines reported similar results [17,31]. On the other hand, total sugars were positive and significantly correlated with phenolics, flavonoids and RAC. Pirie and Mullins [32] reported a good correlation in grapes between sugar content in berries and levels of phenolic substances, probably due to the role of sugars in the regulation of phenolic biosynthesis. Similarly, Abidi *et al.* [19] reported a positive correlation between total sugars and total phenolics, vitamin C and RAC in nectarines. Total sugars, phenolics and flavonoids contents showed a slight significant positive correlation with fruit weight and SSC, showing a tendency of bigger and sweeter fruits to have higher levels of these bioactive compounds. The relationship of fruit weight and SSC with bioactive compounds could be explained by the well-known influence of the sink size on the ability to attract photosynthates from the plant sources, because a sufficient accumulation of sugars near the fruit is essential for phenolic compounds synthesis during fruit growth [33]. Thus, rootstocks inducing bigger and sweeter fruits could be also producing fruits with higher content on antioxidant compounds, as the Pollizo Adesoto rootstock. The correlations between SSC and individual and total sugars were also significant, as previously reported in peaches and nectarines [17,34] or in apricots [16]. A positive correlation between SSC and phenolics content, flavonoids and RAC was also found in other studies with peaches and nectarines [18,19], apricots [35] and sweet cherries [36].

Moreover, we found significant positive correlations between relative antioxidant capacity and total phenolics, flavonoids, and vitamin C, and between vitamin C and phenolics and flavonoids. Thus, flavonoids and total phenolics contribute significantly to the antioxidant capacity of fruits as reported in peaches [18,19,37] or in cherries [36]. Gardner *et al.* [38] showed the contribution of vitamin C to the antioxidant capacity of fruit juices. These results showed that phenolic acids and flavonoid compounds are the main source of antioxidants in fruits [37,39]. However, no significant correlation was obtained between anthocyanins and RAC in our study, as reported by Cantín *et al.* [18] in peaches and nectarines, probably due to their lower content compared with strawberries, raspberries and plums [37]. The high positive correlation found between total phenolics and flavonoids content, indicates that flavonoids are an important group of phenolic compounds in peaches and nectarines with high antioxidant activity.

### 2.3. Principal Component Analysis (PCA) for Agronomical Parameters, Fruit Sugar Content and Phytochemical Traits

**Figure 1.**
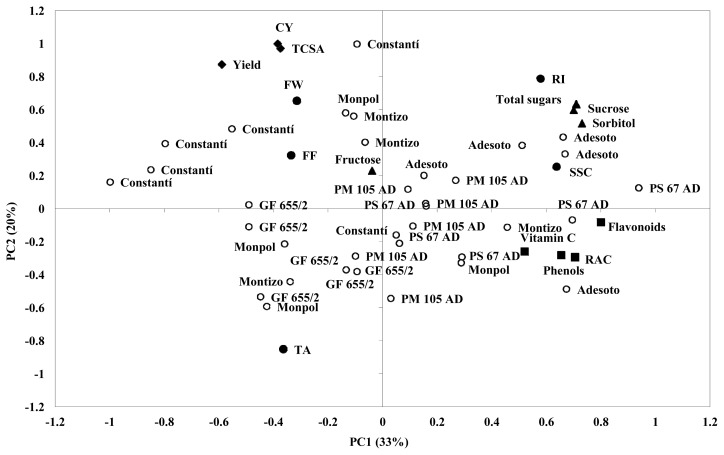
Principal components analysis for the agronomic, basic fruit quality and phytochemical traits evaluated on different plum rootstocks budded with “Catherine” peach cultivar. Analysis was performed using mean data of the three years of study (2009–2011). Symbols: (◆) agronomical traits; (●) basic quality fruit traits; (▲) sugars; (■) antioxidants compounds; and (○) studied rootstocks.

A principal component analysis (PCA) was performed to understand how agronomical and fruit quality traits contribute to variability among the different rootstocks budded with “Catherine” peach cultivar (Figure 1). The first two PCs (PC1 and PC2) accounted for more than 50% of the total variance. PC1 represented the 33% of the variance and PC2 showed the 20% of the variance (Table 6).

The distribution of individuals based on the PC1 and PC2 shows the phenotypic variation and how widely dispersed they are along axes. The PC1 represents mainly SSC, phenolic content, flavonoids, RAC, vitamin C, sucrose, sorbitol and total sugars. The PC2 explains mainly TCSA, yield, cumulative yield, fruit weight, TA, RI and fructose. The results of the analysis of PCA show that trees on the negative side of PC1 and PC2 corresponding to GF 655/2 rootstock, induced lower TCSA, yield and cumulative yield. Trees on the positive side of PC1 corresponding to Adesoto and PM 105 AD rootstock, showed in general higher values of fruit quality traits, such as SSC, sucrose, sorbitol and total sugars. Also, some trees corresponding to Adesoto and P. Soto 67 AD rootstock had higher values on several phytochemical compounds, such as vitamin C, phenols, RAC and flavonoids. The rest of the trees corresponding to Constantí 1 and Monpol had lower or medium values on phytochemical compounds.

The results obtained with the PCA confirm that the rootstock Adesoto induced the higher values on sugar profile (individuals and total sugars) and phytochemical compounds of the “Catherine” peach cultivar, in agreement with Orazem *et al.* [7] and Orazem *et al.* [25].

## 3. Experimental

### 3.1. Plant Material and Field Trial

Seven plum rootstocks (Table 7), including five Pollizo plums (*Prunus insititia*): Adesoto, Monpol, Montizo, P. Soto 67 AD and PM 105 AD, a St. Julien plum (*P. insititia*): GF 655/2, and a common local plum (*P. domestica*): Constantí 1, were evaluated for three consecutive years (2009–2011).

The seven rootstocks were budded with “Catherina” peach cultivar during the summer of 1997, and trees were established in a trial during the winter of 1998–1999. Adesoto (formerly Adesoto 101) and PM 105 AD [22,40] were selected as polyvalent clonal rootstocks for different *Prunus* species, but especially for peaches to avoid water-logging and iron chlorosis in heavy and calcareous soils. Constantí 1 is a local autochthonous plum that has shown a good performance as peach rootstock in field trials at the Experimental Station of Aula Dei [21,41]. Montizo and Monpol are also two Pollizo clonal selections from the “Centro de Investigación y Tecnología Agroalimentaria de Aragón” (CITA, Zaragoza, Spain) [42]. St. Julien GF 655/2 was a rootstock selection developed at the “Institut National de la Recherche Agronomique” (INRA, Bordeaux, France) [43].

The experiment was located at the Experimental Station of Aula Dei (CSIC, Zaragoza, Spain), on a heavy and calcareous soil, with 30.5% total calcium carbonate, 8.8% active lime, water pH 7.7, and a clay-loam texture. Trees were trained to a low-density open-vase system (5 × 4 m). Cultural management practices, such as fertilization, winter pruning, and spring thinning, were conducted as in a commercial orchard. Open vase trees were pruned to strengthen existing scaffold branches and eliminate vigorous shoots, inside and outside the vase, that would compete with selected scaffolds or shade fruiting wood. Moderate-sized fruiting wood (0.3–0.6 m long) was selected. Trees were hand-thinned at 45–50 days after full bloom (DAFB) leaving approximately 20 cm between fruits. The plot was level-basin irrigated every 12 days during the summer. Guard rows were used to preclude edge effects. The experiment was established in a randomized block design with six replications for each scion-stock combination except for Adesoto with five replications. All trees budded on Adesoto and PM 105 AD survived well to the end of the experiment. In contrast, a rate of 33% of tree mortality was found on Montizo and Monpol. Lower mortality was found for P. Soto 67 AD with only a single dead tree (16.6%). Consequently, six replications (trees) were used for each rootstock and year except for Adesoto and PS 67 AD, with five replications; and Monpol and Montizo with only four replications.

### 3.2. Fruit Sampling and Evaluation of Agronomic, Sugar and Phytochemical Traits

Twenty mature fruits of each tree were randomly selected at harvest. The mean fruit weight was calculated considering the total number of fruits and the total yield per tree. The trunk cross-sectional area (TCSA), yield and yield efficiency were also calculated for each scion-stock combination as previously reported [5]. In the entire fruits, values of L* (brightness or lightness), a* (−a* = greenness, +a* = redness), b* (−b* = blueness, +b* = yellowness), C* (chroma) and H (lightness’s angle) were measured using a colorimeter (Chroma Meter, CR-400 Konica Minolta, Tokyo, Japan). Flesh firmness (N) was measured on two paired sides of each fruit, by removing a 1 mm thick disk of skin from each side of the fruit, and using a penetrometer (Model FT-327, QA Supplies, Norfolk, VA, USA). After skin colour and flesh firmness determinations, the fruits of the sample were peeled, and a portion of the mesocarp was removed from each opposite face and cut into small pieces. A composite sample was built by mixing all pieces from all the selected fruits and soluble solids content (SSC) of fruit juice was measured with a digital refractometer (Atago PR-101, Tokyo, Japan), and was expressed as °Brix. The titratable acidity (TA) of samples was determined using an automatic titrator (862 Compact Titrosampler, Metrohm, Herisau, Switzerland). Five grams of homogenized samples were diluted with 45 g of distilled water, and microtitrated with 0.1 N NaOH, and was expressed as g malic acid/100 g FW. Ripening index was calculated based on the SSC/acidity ratio.

For sugars analysis, a composite sample of 5 g was frozen in liquid nitrogen and kept at −20 °C until analyzed. Samples were homogenized with 10 mL of extraction solution consisting of 800 mL/L ethanol/Milli-Q water. The mixture was centrifuged at 20,000 *g* for 20 min at 4 °C. For the analysis, 250 μL of the homogenized extract was incubated at 80 °C for 20 min in 200 μL of 800 mL/L ethanol/water, with 5 g/L manitol added as an internal standard. Samples were purified using ion exchange resins (Bio-Rad, Barcelona, Spain) as reported by Moing *et al.* [45]. Samples were then vacuum concentrated and then resuspended to 1 mL of Milli-Q water (EMD Millipore Corporation, Billerica, MA, USA), before HPLC analysis. The most important sugars found in fruit flesh (sucrose, glucose, fructose and sorbitol) were analyzed by HPLC (Aminex HPX-87C column, 300 mm × 7.8 mm; Bio-Rad, Barcelona, Spain) with a refractive index detector (Waters 2410, Waters Corporation, Milford, MA, USA) as described by Cantín et al. [17]. PC Millenium 3.2 software (Waters Corporation, Milford, MA, USA) was used to perform sugar quantification. A distilled deionized water solution was used as mobile phase with a flow rate of 0.6 mL/min at 85 °C. HPLC peaks were identified using commercial standards of analytical grade (Panreac Quimica SLU, Barcelona, Spain) and standard calibration curves were used to quantify different soluble sugars. Sugar concentrations were expressed as g per kg of fresh weight.

For phytochemical analysis, a composite sample of 5 g was frozen in liquid nitrogen and kept at −20 °C until analyzed. Samples were homogenized in a polytron (T25D Ultra-Turrax, IKA Works Inc., Wilmington, NC, USA) with 10 mL of extraction solution consisting of 0.5 N HCl in methanol/Milli-Q water (80% *v*/*v*). Extracts were centrifuged at 20,000 *g* for 20 min at 4 °C, and the supernatant was collected and stored at −20 °C. The antioxidant compounds were analyzed using a spectrophotometer photodiode array detector DU 800 (Beckman Coulter, Inc., Fullerton, CA, USA) as described by Cantín *et al.* [18]. Standard calibration curves were daily prepared. The Folin-Ciocalteau reagent (Sigma-Aldrich, Steinheim, Germany) at 0.25 N was used to determine the total phenolics content. Absorbance was measured at 725 nm and the results were expressed as mg of gallic acid (3,4,5-trihydroxy-benzoic acid) equivalents (GAE) per 100 g FW. The flavonoid content absorbance was measured at 510 nm and the results were expressed as mg of catechin equivalents per 100 g of FW. For determining anthocyanin content, spectrophotometric readings at 535 nm were taken subtracting absorbance at 700 nm (due to turbidity). Anthocyanins were expressed as mg of cyaniding 3-glucoside equivalents (C3GE) per kg of FW using a molecular weight of 494 and a molar extinction absorptivity coefficient ɛ = 25,965/cm M. The relative antioxidant capacity (RAC) was determined using the 1,1-diphenyl-2-picrylhydrazyl (DPPH). Absorbance was measured at 515 nm and the results were expressed as μg of Trolox equivalents per g of FW. Samples for vitamin C determination were kept at −20 °C in metaphosphoric solution (5% metaphosphoric acid) until analysis for preservation of oxidation. Samples were homogenized with 5% metaphosphoric acid and then centrifuged at 20,000 *g* for 15 min at 4 °C, and the supernatant stored at −20 °C. Absorbance for vitamin C was determined at 525 nm and the results were expressed as mg of ascorbic acid (AsA) per 100 g of FW.

### 3.3. Data Analysis

The data of the means from six replicates were analyzed statistically using SPSS 19.0 (SPSS, Inc, Chicago, IL, USA) and evaluated by ANOVA analysis. When the *F* test was significant, means were separated by Duncan’s multiple range (*p* ≤ 0.05). Data were analyzed to determine the significance of differences between rootstocks. The analyses of Pearson correlation and principal components analysis (PCA) were carried out to study correlations among agronomical, fruit quality, sugars content and phytochemical constituents. A 2D PCA plot was designed using combined data from three years of the fruit quality evaluation and twelve years of the tree agronomical performance using the program Unscrambler version 9.6 program package (Camo, Oslo, Norway).

## 4. Conclusions

The results of this study demonstrate that fruit quality and phytochemical characteristics of peach fruits were significantly affected by rootstocks and those parameters should become more important to be considered in new plantings. The plum rootstock Adesoto seem to induce higher sweetness to peach fruits, based on specific and total sugars and SSC, as well as higher content on antioxidant compounds, based on total phenolics, flavonoids, vitamin C and relative antioxidant capacity. In addition, the results of this work show the importance of the sugar profile, because specific sugars play an important role in peach flavour quality, as well as the studied phytochemical compounds to be considered in looking for high quality peaches with enhanced health properties.

## Figures and Tables

**Table 1.** ANOVA analysis of the effect of rootstock and year on agronomic and fruit quality traits in “Catherine” cultivar for the average of the years of study.

Source of variation 1	Rootstock	Year	Rootstock *x*Year
Trunk cross-sectional area (TCSA)	***	***	ns
Yield	***	***	ns
Cumulative yield	**	**	ns
Yield efficiency	*	**	ns
Fruit weight (FW)	***	***	ns
Soluble solid content (SSC)	***	***	ns
Flesh firmness (FF)	***	***	ns
Titratable acidity (TA)	ns	***	ns
Ripening index (RI)	***	***	ns
Sucrose	**	ns	ns
Glucose	ns	***	ns
Fructose	*	***	ns
Sorbitol	***	***	ns
Total sugars	***	ns	ns
Phenolics	***	**	ns
Flavonoids	***	ns	ns
Anthocyanins	ns	***	ns
Vitamin C	***	ns	ns
Relative antioxidant capacity (RAC)	***	ns	ns

1 Data were evaluated by two-way variance (ANOVA);

*** *p* ≤ 0.001;

** *p* ≤ 0.01;

* *p* ≤ 0.05;

ns, not significant.

**Table 2.** Influence of different plum rootstocks on fruit weight, soluble solids content (SSC), flesh firmness and ripening index of “Catherine” peach fruits in the tenth (2009), eleventh (2010) and twelfth (2011) year after budding.

Trait	Rootstock	2009	2010	2011	Average
Fruit weight (g)	Adesoto	164.4 ab	176.2 ab	179.3 bc	173.3 bc
	Monpol	154.4 a	169.2 a	171.2 ab	165.0 ab
	Montizo	162.2 ab	170.1 ab	162.9 ab	165.1 ab
	P. Soto 67 AD	156.2 a	167.8 a	167.6 ab	163.8 a
	PM 105 AD	154.3 a	164.3 a	171.7 ab	163.4 a
	GF 655/2	163.8 ab	172.2 ab	157.5 a	164.1 a
	Constantí 1	169.5 b	180.7 b	181.5 c	177.2 c

SSC (°Brix)	Adesoto	14.1 ab	13.6 a	13.0 bc	13.5 bc
	Monpol	14.2 ab	13.6 a	12.7 ab	13.5 bc
	Montizo	13.4 ab	12.3 a	11.8 a	12.5 a
	P. Soto 67 AD	14.1 ab	13.4 a	12.1 ab	13.3 b
	PM 105 AD	14.6 b	13.7 a	13.3 c	13.9 c
	GF 655/2	12.8 a	12.8 a	12.2 ab	12.6 a
	Constantí 1	13.4 ab	12.2 a	12.5 ab	12.7 ab

Flesh firmness (N)	Adesoto	32.0 ab	31.0 a	26.2 a	29.7 a
	Monpol	32.0 ab	33.9 ab	26.7 a	30.9 a
	Montizo	35.7 b	37.5 b	20.0 a	31.1 a
	P. Soto 67 AD	33.7 ab	35.1 ab	30.8 a	33.2 a
	PM 105 AD	30.9 ab	31.6 a	25.0 a	29.1 a
	GF 655/2	29.8 a	32.5 a	28.7 a	30.3 a
	Constantí 1	32.9 ab	35.2 ab	26.6 a	31.6 a

Ripening index (SSC/TA)	Adesoto	31.6 b	23.1 a	21.1 a	25.3 b
	Monpol	29.0 ab	22.8 a	20.3 a	26.5 b
	Montizo	24.5 a	20.6 a	18.5 a	23.7 a
	P. Soto 67 AD	29.0 ab	22.4 a	19.6 a	24.0 ab
	PM 105 AD	29.2 ab	22.5 a	20.5 a	24.1 ab
	GF 655/2	23.7 a	19.4 a	17.5 a	20.2 a
	Constantí 1	26.5 ab	20.5 a	19.6 a	22.2 a

a, b, or c are used to indicate statistically significant differences among means when comparing more than two groups (multiple means separation). For each year and trait, means followed by the same letter in each column are not significantly different at *p* ≤ 0.05 according to Duncan’s Multiple Range Test.

**Table 3.** Influence of different plum rootstocks on the individual and total sugars of “Catherine” peach fruits in the tenth (2009), eleventh (2010) and twelfth (2011) year after budding.

Trait	Rootstock	2009	2010	2011	Average
Sucrose	Adesoto	69.9 a	72.1 b	70.2 b	70.7 b
	Monpol	66.9 a	65.0 a	66.5 ab	66.1 a
	Montizo	61.0 a	62.3 ab	67.2 ab	63.5 a
	P. Soto 67 AD	69.0 a	66.9 ab	61.2 a	65.7 a
	PM 105 AD	68.5 a	67.6 ab	67.6 ab	67.9 ab
	GF 655/2	63.0 a	67.0 ab	61.9 ab	64.0 a
	Constantí 1	62.5 a	63.6 ab	66.8 ab	64.3 a

Glucose	Adesoto	7.9 a	8.5 a	7.2 a	7.9 a
	Monpol	9.0 a	9.0 a	8.3 a	8.8 a
	Montizo	9.2 a	9.2 a	8.1 a	8.8 a
	P. Soto 67 AD	8.9 a	8.5 a	7.3 a	8.2 a
	PM 105 AD	8.7 a	8.2 a	7.6 a	8.2 a
	GF 655/2	8.4 a	8.6 a	7.3 a	8.1 a
	Constantí 1	8.5 a	8.8 a	7.7 a	8.3 a

Fructose	Adesoto	10.2 b	9.8 a	9.3 b	9.8 b
	Monpol	9.4 ab	9.1 a	8.5 ab	9.0 ab
	Montizo	9.8 ab	10.1 a	9.0 ab	9.4 ab
	P. Soto 67 AD	9.8 ab	9.7 a	8.5 ab	9.3 ab
	PM 105 AD	8.8 a	9.0 a	8.2 a	8.7 a
	GF 655/2	9.4 ab	10.1 a	8.6 ab	9.4 ab
	Constantí 1	9.6 ab	9.8 a	8.7 ab	9.4 ab

Sorbitol	Adesoto	6.2 b	5.8 b	4.8 b	5.6 b
	Monpol	4.4 a	4.5 a	4.0 ab	4.3 ab
	Montizo	3.8 a	3.9 a	3.1 a	3.6 a
	P. Soto 67 AD	4.7 a	4.7 ab	3.5 ab	4.3 ab
	PM 105 AD	5.1 a	4.8 ab	4.2 ab	4.7 ab
	GF 655/2	3.7 a	4.1 a	3.0 a	3.6 a
	Constantí 1	3.6 a	3.6 a	3.7 ab	3.6 a

Total sugars	Adesoto	94.2 b	96.2 b	91.5 b	94.0 b
	Monpol	89.7 ab	87.6 ab	87.3 ab	88.2 ab
	Montizo	83.8 a	85.5 a	87.4 ab	85.5 a
	P. Soto 67 AD	92.4 ab	89.8 ab	80.5 a	87.5 a
	PM 105 AD	91.1 ab	89.6 ab	87.6 ab	89.5 ab
	GF 655/2	84.5 a	89.8 ab	80.8 a	85.1 a
	Constantí 1	84.2 a	85.8 a	86.9 ab	85.6 a

For each year, means followed by the same letter in each column are not significantly different at *p* ≤ 0.05 according to Duncan’s Multiple Range Test. All individual sugars and total sugars (g kg^−1^ FW).

**Table 4.** Influence of different plum rootstocks on the antioxidant compounds of “Catherine” peach fruits in the tenth (2009), eleventh (2010) and twelfth (2011) year after budding.

Trait	Rootstock	2009	2010	2011	Average
Total phenolics	Adesoto	33.9 b	31.8 c	34.3 c	33.4 c
	Monpol	25.0 a	24.5 ab	28.7 ab	26.1 ab
	Montizo	28.2 a	26.7 bc	28.7 ab	28.0 b
	P. Soto 67 AD	28.5 a	28.0 bc	30.7 bc	28.9 bc
	PM 105 AD	28.8 a	29.7 bc	29.1 ab	29.2 bc
	GF 655/2	26.2 a	25.9 ab	29.9 bc	27.4 b
	Constantí 1	24.2 a	22.8 a	26.0 a	24.3 a

Flavonoids	Adesoto	11.7 c	10.2 bc	10.2 c	10.7 c
	Monpol	7.7 ab	8.1 ab	8.2 ab	8.0 ab
	Montizo	9.9 bc	9.7 bc	8.8 bc	9.5 bc
	P. Soto 67 AD	9.9 bc	9.0 bc	8.2 bc	9.1 ab
	PM 105 AD	10.9 c	11.1 c	9.6 bc	10.6 bc
	GF 655/2	7.7 ab	7.7 ab	7.9 ab	7.8 a
	Constantí 1	7.4 a	6.6 a	7.0 a	7.0 a

Anthocyanins	Adesoto	0.46 a	0.47 a	0.59 a	0.50 a
	Monpol	0.49 a	0.53 a	0.65 a	0.56 a
	Montizo	0.39 a	0.44 a	0.63 a	0.49 a
	P. Soto 67 AD	0.40 a	0.41 a	0.62 a	0.47 a
	PM 105 AD	0.46 a	0.47 a	0.66 a	0.53 a
	GF 655/2	0.47 a	0.46 a	0.64 a	0.52 a
	Constantí 1	0.45 a	0.50 a	0.62 a	0.52 a

Vitamin C	Adesoto	8.9 c	9.1 b	8.6 b	8.8 c
	Monpol	7.4 b	6.9 a	7.6 a	7.3 b
	Montizo	8.0 bc	8.1 ab	8.0 ab	8.0 bc
	P. Soto 67 AD	7.3 bc	7.8 ab	7.4 a	7.5 b
	PM 105 AD	9.0 c	9.6 b	8.8 b	9.1 c
	GF 655/2	5.9 ab	6.2 a	5.6 a	5.9 a
	Constantí 1	5.4 a	6.1 a	5.8 a	5.7 a

RAC	Adesoto	502.2 c	466.7 b	430.0 b	466.3 c
	Monpol	434.5 bc	416.2 ab	414.4 ab	421.7 bc
	Montizo	410.3 ab	398.1 ab	382.9 ab	397.1 ab
	P. Soto 67 AD	436.8 bc	415.5 ab	383.0 ab	413.8 ab
	PM 105 AD	429.0 bc	440.4 b	406.0 ab	425.2 bc
	GF 655/2	413.1 ab	411.9 ab	401.3 ab	408.8 ab
	Constantí 1	345.6 a	350.3 a	358.1 a	351.3 a

For each year, means followed by the same letter in each column are not significantly different at *p* ≤ 0.05 according to Duncan’s Multiple Range Test. Total phenolics (mg GAE/100 g FW); flavonoids (mg CE/100 g FW); anthocyanins (mg C3GE/kg FW); vitamin C (mg AsA/100 g FW); RAC, relative antioxidant capacity (μg Trolox/g FW).

**Table 5.** Pearson’s correlations coefficients between some agronomical, basic fruit quality and phytochemical traits on different plum rootstocks budded with “Catherine” cultivar.

Trait	TCSA	YE	FW	SSC	Sucrose	Glucose	Fructose	Sorbitol	TS ^a^	Phenolics	Flavonoids	Anthocyanins	Vitamin C	RAC
Yield	0.63 **	0.36 *	0.36 **	−0.39 **	−0.52 **	ns	ns	ns	ns	ns	−0.35 **	0.40 **	−0.28 **	−0.35 *
YE		-	ns	ns	ns	−0.36 *	ns	ns	ns	ns	ns	ns	ns	ns
FW			-	0.36 **	0.42 **	0.54 **	ns	ns	0.37 **	0.26 **	0.30 **	ns	ns	ns
SSC				-	0.41 **	0.52 **	0.39 *	0.43 **	0.43 **	0.37 **	0.32 **	ns	ns	0.29 **
Sucrose					-	0.65 *	0.63 **	0.52 **	0.85 *	ns	ns	ns	ns	ns
Glucose						-	0.56 **	0.64 **	0.98 *	ns	ns	ns	ns	ns
Fructose							-	0.45 *	0.42 **	ns	ns	ns	ns	ns
Sorbitol								-	0.41 *	ns	ns	ns	ns	ns
TS ^a^									-	0.53 **	0.46 **	ns	ns	0.37 **
Phenolics										-	0.71 **	ns	0.33 **	0.52 **
Flavonoids											-	ns	0.36 **	0.65 **
Anthocyanins												-	ns	ns
Vitamin C													-	0.25 **

* and ** represent statistical significance at *p* ≤ 0.05 and *p* ≤ 0.01 respectively; ns, not significant. Abbreviations: TCSA, trunk cross-sectional area; YE, yield efficiency; FW, fruit weight; SSC, soluble solid content; TS ^a^, Total sugars, the sum of sucrose, glucose, fructose, and sorbitol for each genotype, analyzed by HPLC and RAC, relative antioxidant capacity.

**Table 6.** Eigenvectors of the principal component analysis (PCA) axes of the agronomical, basic fruit quality and phytochemical traits evaluated on different plum rootstocks budded with “Catherine” cultivar.

Traits	Component loading	

	PC1 (33%)	PC2 (20%)
Trunk cross-sectional area (TCSA)	−0.374	0.972
Yield	−0.59	0.875
Cumulative yield (CY)	−0.384	1
Fruit weight (FW)	−0.314	0.654
Soluble solid content (SSC)	0.637	0.255
Flesh firmness (FF)	−0.336	0.325
Titratable acidity (TA)	−0.365	−0.852
Ripening index (RI)	0.579	0.788
Phenolic content	0.654	−0.28
Flavonoids	0.8	−0.0812
Relative antioxidant capacity (RAC)	0.705	−0.294
Vitamin C	0.52	−0.259
Sucrose	0.7	0.598
Fructose	−0.0392	0.23
Sorbitol	0.73	0.517
Total sugars	0.71	0.635

**Table 7.** List of studied rootstocks, description and origin.

Rootstock	Species	Genetic background	Origin a	References
Adesoto b	*P. insititia*	op d Pollizo, clonal selection	CSIC, Spain	[22,40]
Monpol	*P. insititia*	op d Pollizo, clonal selection	CITA, Spain	[42]
Montizo	*P. insititia*	op d Pollizo, clonal selection	CITA, Spain	[42]
P. Soto 67 AD c	*P. insititia*	op d Pollizo	CSIC, Spain	[40,44]
PM 105 AD c	*P. insititia*	op d Pollizo, clonal selection	CSIC, Spain	[40]
GF 655/2	*P. insititia*	St. Julien clonal selection	INRA, France	[43]
Constantí 1 c	*P. domestica*	op d, common local plum	CSIC, Spain	[21,41]

a CSIC = Consejo Superior de Investigaciones Científicas; CITA = Centro de Investigación y Tecnología Agroalimentaria de Aragón; INRA = Institut National de la Recherche Agronomique;

b Protected grant by Community Plant Variety Office (CPVO);

c non-released clones from the Aula Dei breeding program; and

d op: open-pollinated.
